# Crosstalk Between Autophagy and Inflammation in Chronic Cerebral Ischaemia

**DOI:** 10.1007/s10571-023-01336-6

**Published:** 2023-03-23

**Authors:** Hai-qian Zhou, Li-mei Zhang, Xiao Li, Zhi-hua Huang

**Affiliations:** 1 Key Laboratory of Prevention and Treatment of Cardiovascular and Cerebrovascular Diseases of Ministry of Education, Gannan Medical University, 1st Hexie Road, Ganzhou, 341000 China; 2 Department of Physiology, School of Basic Medical Sciences, Gannan Medical University, 1st Hexie Road, Ganzhou, 341000 China; 3 Ganzhou Key Laboratory of Neuroinflammation Research, Gannan Medical University, 1st Hexie Road, Ganzhou, 341000 China

**Keywords:** Autophagy, Inflammation, Chronic cerebral ischaemia

## Abstract

Chronic cerebral ischaemia (CCI) is a high-incidence cardiovascular and cerebrovascular disease that is very common in clinical practice. Although many pathogenic mechanisms have been explored, there is still great controversy among neuroscientists regarding the pathogenesis of CCI. Therefore, it is important to elucidate the mechanisms of CCI occurrence and progression for the prevention and treatment of ischaemic cerebrovascular disorders. Autophagy and inflammation play vital roles in CCI, but the relationship between these two processes in this disease remains unknown. Here, we review the progression and discuss the functions, actions and pathways of autophagy and inflammation in CCI, including a comprehensive view of the transition from acute disease to CCI through ischaemic repair mechanisms. This review may provide a reference for future research and treatment of CCI.

**Graphical Abstract:**

*Schematic diagram of the interplay between autophagy and inflammation in CCI*. CCI lead to serious, life-threatening complications. This review summarizes two factors in CCI, including autophagy and inflammation, which have been focused for the mechanisms of CCI. In short, the possible points of intersection are shown in the illustration. CCI, Chronic cerebral ischaemia; ER stress, Endoplasmic reticulum stress; ROS, Reactive oxygen species.
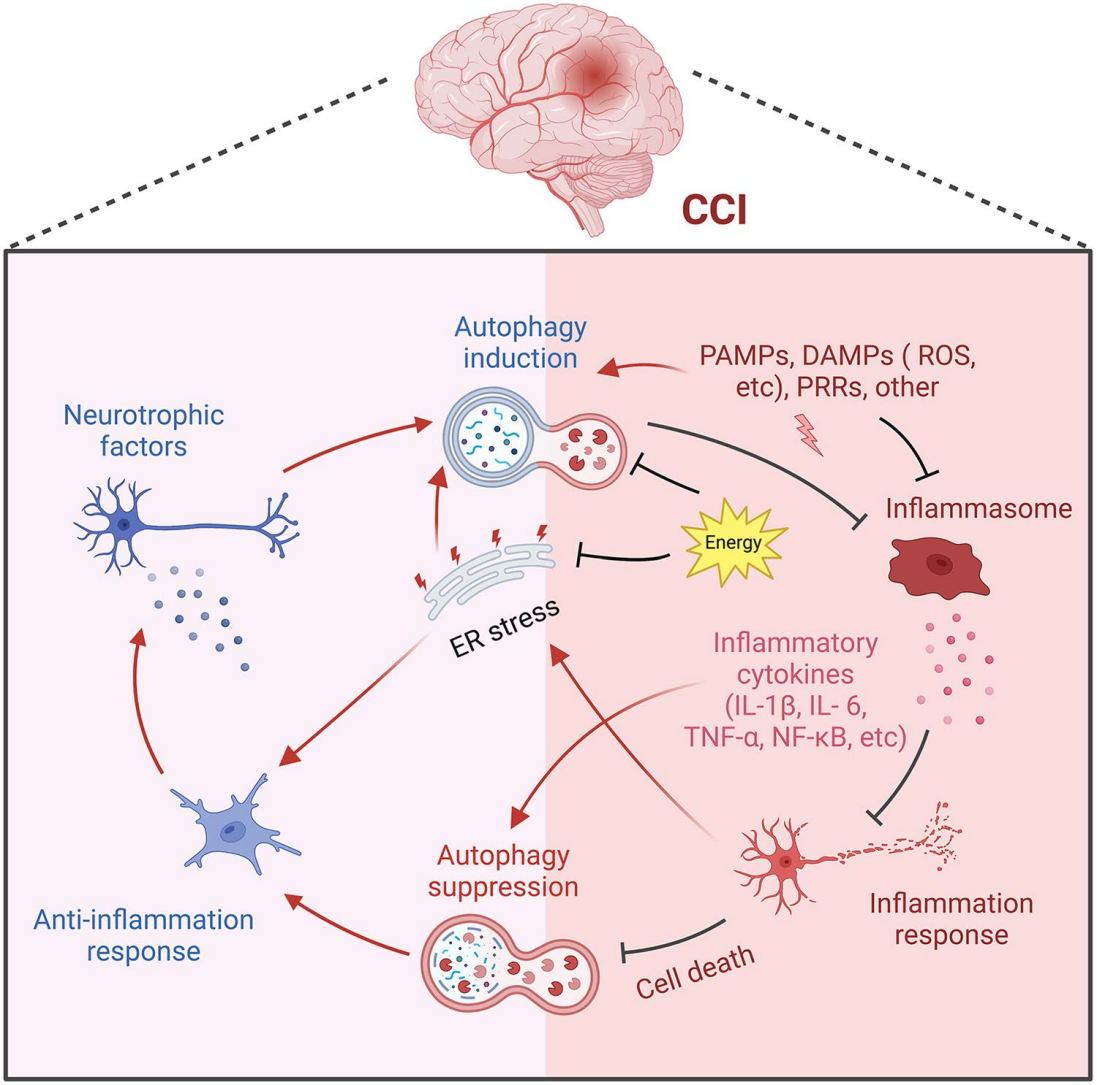

## Introduction

Ischemic cerebrovascular disease is mainly caused by system hypoperfusion, cerebral vascular stenosis, thrombosis or embolism (Caplan 2006), including cerebral infarction (CI) and chronic cerebral ischaemia (CCI). CI is also called ischemic stroke. It occurs when a blood vessel in the brain becomes blocked (Sherman et al. 2021), which leads to hypoxic-ischemic necrosis of brain tissue, resulting in clinically corresponding neurological defects. CCI, which mostly occurs in elderly individuals, refers to a decrease in the overall blood supply to the brain (Alia et al. 2017). When this condition is not cured, a series of pathological changes can be observed in brain tissue over time, such as changes in glial activity, inflammation and the degeneration of neurons (Du et al. 2017). The pathological process of CCI damage is complicated and includes excitotoxicity, calcium excess, oxidative stress, cell death, necrosis, autophagy, and inflammation.

Inflammation is a protective reaction in the body and an immune response in tissues to various stimuli, including microbial pathogens and endogenous molecules. However, long-term chronic inflammation can cause damage due to high concentrations of inflammatory mediators in exposed tissues. As a vital mechanism related to the pathophysiological process, inflammation has an important function in the onset and progression of CCI (Deng et al. 2018; Yang et al. 2017b). An FDA-approved medicine alleviates spatial memory deficits in CCH rats, which may be linked to anti-inflammatory impacts (Yan et al. 2021). By taking part in the disposal of injured organelles and denatured proteins and degrading pathogens through the lysosomal pathway, autophagy functions to preserve homeostasis in cells. Increasing evidence has revealed that autophagy also plays a role in CCI (Wang et al. 2017).

Autophagy and inflammation are considered to be interdependent processes. Recently, autophagy-dependent mechanisms have been associated with the onset of a number of inflammatory disorders, including infectious disorders, Crohn’s disease, pulmonary hypertension, and malignancy (Ceccariglia et al. 2020; Cosin-Roger et al. 2017; Lee et al. 2018; Schlemmer et al. 2018; Sorbara et al. 2013; Tang et al. 2018). These studies indicate that there are some unclear crosstalk mechanisms between autophagy and the inflammatory signalling cascade. Although some progress has been made in elucidating the function of autophagy in inflammation, the molecular mechanism of the reaction between autophagy and inflammation in CCI are still relatively poorly understood. Inflammation and autophagy may be critical players in CCI, which causes neuronal loss and vascular cognitive impairment (Wang et al. 2020). In this review, we analysed the connection between autophagy and inflammation in the pathogenesis of CCI to provide novel insights into CCI.

## Relationship Between Inflammation and CCI

### Inflammation is Involved in the Repair Process of CCI

During cerebral ischaemia, the brain responds to the injury and triggers an inflammatory cascade response by releasing harmful materials, particularly necrotic cell debris, which lead to not only subsequent tissue injury but also the repair process (Li et al. 2022). In addition, subsequent regeneration can be triggered in tissues with secondary injury signals, such as free radicals and inflammatory cytokines (Carmichael 2016). This finding shows that damage and repair processes occur not only in the local stroke site but also in the neuronal connection network at the stroke site. This means that damage signals can be transmitted through the brain network. These transmitted injury signals can increase reactive astrocytes and inflammation in distant brain regions, leading to the formation of new connections. The inflammatory cytokine TNF is a valuable and useful neuroprotective factor in the acute stage, and it may serve as a regulator of neuroinflammation after cerebral ischaemia, which is beneficial in the recovery of cerebral ischaemia (Clausen et al. 2016), suggesting that inflammation is one of the repair mechanisms of CCI.

### Inflammation Involved in CCI Injury

Recently, evidence has shown that stroke infarcts are zones of chronic inflammation (Zbesko et al. 2018). Important variables that influence the onset and development of inflammatory responses are inflammatory mediators. Multiple inflammatory mediators have been revealed to play significant roles in the inflammatory reaction to CCI. The chronic phase is the main stage of cognitive dysfunction caused by chronic cerebral hypoperfusion (CCH). CCH is associated with highly activated inflammatory pathways and inflammation-mediated effects, which induce neuroinflammation and lead to white matter lesions, neuronal loss, and learning and memory disorders. Therefore, we should pay more attention to chronic inflammation in CCH models (Sattayakhom et al. 2022).

Yoshizaki et al. (2008) found that interleukin-1β (IL-1β), interleukin-6 (IL-6), and tumour necrosis factor-α (TNF-α) levels were substantially upregulated in the ischaemic hemisphere at 2 h after surgery in a CCH model, and monocyte chemotactic protein-1 (MCP-1), IL-6, and TNF-α were also detected at 24 h after surgery; the anti-inflammatory cytokines IL-4 and IL-10 were not altered until 3 days following occlusion (Kitamura et al. 2012). After CCI, the level of TNF-α in serum and brain tissue was increased, and the production of IL-6 was also elevated. Inhibiting the production of the proinflammatory cytokines TNF-α and IL-6 may protect against cerebral ischaemia (Pan et al. 2012). These findings reveal that IL-4 and IL-10 deserve attention as anti-inflammatory cytokines in CCI.

The matrix metalloproteinase (MMP) family, which includes important inflammatory mediators, is the most important family in chronic hypoperfusion white matter lesions. MMP-2 and MMP-9 belong to the MMP family, are present in blood vessels, participate in vascular remodelling and have a vital function in the pathophysiology of CCH. Upregulation of MMP-2 and MMP-9 can promote cerebral ischaemia post-functional recovery (Wang et al. 2012). This effect may occur because MMP-2 promotes angiogenesis and compensation during CCH (Corbin et al. 2014).

Inflammation, as measured by VCAM-1 levels, may have an impact on poststroke cognitive problems (El Husseini et al. 2020). In addition, intercellular cell adhesion molecule-1 (ICAM-1) and vascular cell adhesion molecule-1 (VCAM-1) are substantially increased in cerebral vascular endothelial cells in a permanent bilateral vessel occlusion (2-VOS) animal model, and the upregulation of VCAM-1 expression is the initial link between the inflammatory response and adhesion, suggesting that CCI can induce a microvascular inflammatory response and participate in CCI-induced cognitive impairment (Huang et al. 2010).

### Activation of Related Inflammatory Pathways

#### TLRs and the NF-κB Signalling Pathway

Toll-like receptors (TLRs), a class of pattern-recognizing receptors, trigger inflammatory reactions and are expressed by astrocytes, microglia, and neurons. In the central nervous system (CNS), TLRs can recognize multiple types of pathogen-associated molecular patterns (PAMPs) or damage-associated molecular patterns (DAMPs) (Kawabori and Yenari 2015). PAMPs can bind to TLRs, activate receptors and initiate an immune response via the signalling cascade, further leading to the expression of effector molecules. The cascade mobilization of Toll/IL-1R (TIR) adapter proteins is primarily comprised MyD88, NF-κB-inducing kinase (NIK), and IκB kinase (IKK), which stimulate the downstream molecule NF-κB to join the nucleus and initiate the generation of proinflammatory cytokines, such as TNF-α, IL-1, and IL-12 (Zhao et al. 2020). TLRs rely on microglia in the ischaemic core area rather than astrocytes to produce ATP-dependent IL-1β in the CNS during cerebral ischaemia (Facci et al. 2014; Hao et al. 2016).

Recent studies have revealed that TLR4 triggers an inflammatory reaction in CCI and has a vital role in initiating glial activation in cerebral ischaemia‒reperfusion damage (Wang et al. 2016a). The inhibition of TLR4-NOX4 signalling significantly reduces the infarct volume after cerebral infarction and reperfusion and improves neurological functional scores, demonstrating that TLR4 represents a possible therapeutic target for CCI (Suzuki et al. 2012). Downregulation of TLR4 and its downstream signalling factor MyD88 has a neuroprotective impact against cerebral ischaemia (Sun et al. 2015). These findings suggest that the TLR4/MyD88/NF-κB may be the main signalling pathway that regulates the glial inflammatory response.

#### MAPK Signalling Pathway

Mitogen-activated protein kinase (MAPK) signalling, consisting of p38, ERK (extracellular signal-regulated kinase) and JNK (N-terminal kinase), plays a crucial role in glial cell activation. In a model of CCH, AMP-activated protein kinase (AMPK) and the Ca(2+)/calmodulin-dependent protein kinase β (CaMKKβ)-dependent signalling pathway, upstream of MAPK, were also implicated in the activation of microglia (Green et al. 2011; Yuan et al. 2017). Hypoxia increased MCP-1 expression, which activated microglia and the p38MAPK/PKC pathways, which are involved in the process of cognitive deficits produced by CCH (Li et al. 2016). Inhibiting p38 MAPK and MAPK2 (MK2) with pharmacological methods could alleviate the activation of microglia and the production of inflammatory cytokines (TNF-α, IL-6 and IL-1β), suggesting that this signalling pathway may be a therapeutic target for neuroinflammation-related diseases (Bhatia et al. 2017). Studies have revealed that attenuating the activation of hippocampal microglia and astrocytes and inhibiting inflammatory mediators and cytokines and their corresponding intracellular signalling pathways (TLR4/MyD88 and p38 MAPK) can significantly alleviate CCI-induced behavioural impairments (Cheng et al. 2021; Lee et al. 2015).

## Autophagy Plays a Role in CCI

Autophagy has a vital function in preserving homeostasis in the cell and may be necessary for neuronal homeostasis, neuronal plasticity, and protein quality management (Smith et al. 2014; Wang et al. 2006). Cell autophagy can be induced by changes in the intracellular environment, such as organelle and cytoplasmic accumulation or damage, and extracellular stimulation, such as starvation, high temperature, hypoxia, and hormone stimulus (Yang and Klionsky 2010). Autophagy is primarily related to the circulation and reprocessing of macromolecular elements in cells, and the removal of foreign substances from the cytoplasm and damaged organelles by the autophagy lysosome system maintains cell function (Burman and Ktistakis 2010). By playing dual roles in cell survival during this process (Ravanan et al. 2017), autophagy is not entirely beneficial to the body (Liu and Levine 2015). Studies have revealed that autophagy plays a significant role in ischaemic brain injury because CCH can lead to hippocampal atrophy, lowering the Bcl-2/Bax ratio, neuronal apoptosis, and enhancement and redistribution of autophagy in rats (Liu et al. 2015).

Autophagy is activated and may play dual roles in cell death or survival during cerebral ischemia. Autophagy not only mediates direct microbial degradation but also exerts other protective mechanisms, such as mediating inflammation by affecting the progression, homeostasis and survival of inflammatory cells (such as macrophages, neutrophils and lymphocytes), which have a key function in inducing and eliminating the source of inflammatory stimuli together with inflammatory signals, or by affecting the transcription, processing, and secretion of various cytokines, thereby reducing the adverse effects of inflammatory responses (Heckmann et al. 2017; Lambelet et al. 2018; Netea-Maier et al. 2016; Pun and Park 2017). On the other hand, numerous pathological pathways, including mitochondrial dysfunction, acidosis, oxidative stress, calcium overload, excitotoxicity, and the inflammatory reaction, are implicated in the onset and progression of cerebral ischaemia reperfusion, resulting in inhibited glutamate accumulation in brain tissue and an increase in impaired cells, which evoke autophagy to varying degrees (Khoshnam et al. 2017). During this process, autophagy may be triggered through multiple-related signalling pathways, such as the AMPK/TSC/mTOR, Beclin 1/BNIP3/SPK2, PI3K/Akt-mTOR, and FoxO/NF-κB pathways (Qi et al. 2014; Sheng and Qin 2015; Yang et al. 2017d). P301L is the tau mutation most frequently observed in patients with frontotemporal dementia, and an interesting study showed that enhancing autophagy could alleviate stroke in elderly rats by modulating P301L-Tau, which reveals that autophagy could be a potential target for improving cognitive impairments in CCI (Yang et al. 2017a). In animal studies (Ma and Ji 2022; Wu et al. 2021; Zhang et al. 2021), targeting autophagy signalling could be a novel treatment approach to prevent and treat CCI. However, determining whether this treatment will be beneficial for humans requires further investigation.

## The Link Between Autophagy and Inflammation in CCI

The above evidence shows that CCI is associated with inflammation and autophagy. DAMPs released in local tissue due to neuronal and axonal injury further recruit and activate leukocytes to induce secondary brain injury (Huber-Lang et al. 2018; Takeuchi and Akira 2010). Intriguingly, autophagy can modulate the production of DAMPs and participate in the inflammatory regulation mediated by PAMPs or DAMPs by some unclear mechanism. On the other hand, PAMPs and DAMPs can activate autophagy through particular mechanisms, whereas autophagy can suppress an excessive inflammatory response via PAMPs and DAMPs and regulate TLR or NLR signalling to restrict tissue damage (Into et al. 2012). This evidence shows that there is an interaction between autophagy and inflammation, which may present a potential link between CCI, autophagy, and inflammation.

### Autophagy Regulates the Inflammatory Response in CCI

Autophagy plays dual regulatory roles in the inflammatory response to CCI. In a moderate manner, autophagy is effective at anti-inflammation by eliminating inflammatory proteins and proinflammatory cytokines (Bussi et al. 2017). In contrast, excessive autophagy promotes inflammatory responses. As shown in a related study, activation of excessive autophagy induced by CCH simultaneously triggers inflammatory responses that further aggravate cognitive impairment (Yang et al. 2014).

Glial cells are widely distributed in the CNS and mainly include astrocytes, microglia and peritubular glia. Astrocyte differentiation is crucial for advanced embryonic brain development processes, along with activating autophagy. Atg5, an autophagy-related gene, regulates the differentiation of astrocytes in the developing mouse cortex. Deficiency in this gene inhibits the production of astrocytes in vitro and in vivo. In contrast, Atg5 overexpression considerably increases the number of astrocytes (Wang et al. 2014). Altogether, these results demonstrate that autophagy may exert positive effects on glial differentiation.

As a type of immune cell that resides in the CNS, triggered microglia are a sign of neuroinflammation. Autophagy could be triggered by both trehalose and rapamycin in BV2 microglia, therefore decreasing proinflammatory cytokine and nitric oxide (NO) induced by LPS and alpha-synuclein. In addition, autophagy alters the phosphorylation of p38 and ERK1/2 MAPKs in BV2 cells, which is necessary for NO generation (Bussi et al. 2017). Hypoxia inducible factor 1α (HIF-1α), an overexpressed factor in CCH, reduces the density of astrocytes and microglia in the cortex and hippocampus, inhibits oxidative stress and inflammation in the brain, and provokes mitochondrial autophagy, which is frequently accompanied by suppression of the mTOR signalling, thereby enhancing neuronal survival in CCH (Gong et al. 2014). This research implies that HIF-1 may govern the status of glial activation and inflammation in CCH via autophagy. It has been found that a lack of autophagy under nutritional shortage may enhance microglial activation and the severity of inflammation (Bray et al. 2022; Li et al. 2019; Plaza-Zabala et al. 2017). Another study revealed that autophagy is a regulator of endothelial cell-leukocyte trafficking machinery that aims to stop physiological inflammation (Reglero-Real et al. 2021). Therefore, autophagy may regulate the neuroinflammatory response by regulating the activation of glia after CCH.

### Inflammation Regulates Autophagy Through Cytokines and ROS

Cytokines and reactive oxygen species (ROS) are inflammatory mediators that regulate autophagy by altering the intracellular environment. Th1 family cytokines (IL-2, TNF-α, etc.) induce autophagy production, while Th2 family cytokines (IL-4, IL-5, IL-6, IL-10, and IL-13) and anti-inflammatory cytokines have inhibitory effects on autophagy (Harris 2011). In addition, IL-1, TGF-β, and IFN-γ are cytokines that can induce autophagy.

PRRs, such as Toll-like receptors (TLRs), NOD-like receptors (NLRs), RIG-I-like receptors, and C-type lectin receptors, can activate primary inflammatory signals and promote autophagy activation by recognizing PAMPs or DAMPs (Liu et al. 2017). With lung ischaemia–reperfusion damage, DAMPs induce autophagy and increase inflammatory reactions by promoting K63-linked TRAF6 ubiquitination and stimulating MAPK and NF-kappa B signalling (Liu et al. 2017).

Xu et al. (2007) demonstrated that TLR ligands not only contribute to the inflammatory process but also trigger autophagy. Autophagy is also induced by the NLR family members nucleotide-binding oligomerization domain 1 (NOD1) and NOD2. Through adaptor kinase receptor interacting protein 2 (RIP2), these molecular signals drive NF-κB and MAPK signalling. Upregulation of NOD2 signalling can provide feedback to inhibit the activation of NOD2-RIP2 signalling and inflammatory bodies by inducing autophagy in alveolar macrophages and negatively regulating lung pulmonary inflammation (Wen et al. 2014). A recent study showed the role of microglia in prolonged cerebral hypoperfusion. Voltage-gated proton channel (Hv1) is expressed in microglia and is involved in the generation of ROS and proinflammatory cytokines in microglia (Yu et al. 2020). The orphan nuclear receptor TLX may exert protective effects on cognitive disorders caused by CCH by regulating the SIRT1/NF-κB pathway (Qu et al. 2021).

This evidence shows that cytokines, ROS, and inflammation-related signalling molecules can activate autophagy through multiple pathways. Therefore, controlling inflammation following CCI is essential for reversing brain injury.

### TLR Signalling Mediates the Interaction Between Autophagy and Inflammation

As the main innate immunity sensors that detect various pathogen-specific molecular patterns, TLRs activate downstream signals mainly through ubiquitin-dependent mechanisms. TLR2 or TLR4 agonists may induce autophagy by supporting the reaction between Beclin1 and MyD88 or TRIF and reducing Beclin1 binding to Bcl-2 (Chuang et al. 2013). Inhibition of the TLR4/MyD88/TRAF6 signalling pathways decreases the inflammatory reaction and autophagy and has a neuroprotective effect on cerebral ischaemia‒reperfusion damage (Chan et al. 2016; Cheng et al. 2019; Nazio et al. 2013; Schneider et al. 2012; Verstak et al. 2014; Wang et al. 2016b).

Selective autophagy degrades TRIF and negatively regulates TLR3/4-mediated natural immune reactions (Shi and Kehrl 2008; Yang et al. 2017c). Another study revealed that basal autophagy reduced the expression of monomeric MyD88, thus preventing the self-activation of inflammatory signals (Into et al. 2017). This evidence partly explains the bidirectional communication between TLR and autophagy. Because TLR activation is present in CCI (Khan et al. 2022; Wang et al. 2016a), further research must be conducted to determine whether it is also related to the excessive stimulation of autophagy in CCI.

### ROS Produced by Mitochondria May be a Link Between Autophagy and Inflammation

A recent study demonstrated that ROS contribute to the progression of CCI. In CCI pathophysiological mechanisms, inflammatory cascades exacerbate mitochondrial damage, causing increased ROS production and the blocking of autophagosome breakdown, thus initiating a vicious cycle (Zeng et al. 2022). As the most abundant organelle, mitochondria generate large amounts of ROS in cells, which regulate the intracellular redox state and energy metabolism (Busija et al. 2016; Ham and Raju 2017). Studies have revealed that the loss of intracellular balance in redox states and the development of free radicals can lead to neuronal damage (Sutkowy et al. 2021), while dysfunctional mitochondria produce ROS (Busija et al. 2016). Under physiological conditions, ROS mediate beneficial responses, while excess ROS are harmful to the body. Especially with I/R, ROS trigger tissue inflammation and activate NLRP3 inflammasomes, thereby inducing neurological dysfunction and inflammatory responses in brain tissue (Minutoli et al. 2016; Zhang et al. 2017).

By limiting the production of ROS, autophagy maintains mitochondrial integrity and inhibits the activation of NALP3-dependent inflammation (Meng et al. 2019; Nakahira et al. 2011). Inhibiting mitochondrial autophagy results in the accumulation of ROS in damaged mitochondria, which in turn triggers the NLRP3 inflammasome (Zhao et al. 2020; Zhou et al. 2011). Therefore, coordinated interactions between autophagy activation and ROS-dependent signalling are necessary for the regulation of innate defence and homeostatic inflammation.

In the cytoplasm of CA1 neurons in CCH rat models, abnormal mitochondrial aggregation and morphological changes have been observed (Hai et al. 2013). ROS-mediated oxidative stress and neuroinflammation are closely correlated with chronic ischaemia‒reperfusion injury in mice (Han et al. 2022), suggesting that mitochondria participate in CCI. Can mitochondria balance cell autophagy and inflammation by increasing or decreasing ROS in CCI? This question has not yet been answered, so further studies are warranted.

## Concluding Remarks

CCI is a major cause of global disability and can lead to permanent tissue damage. The study of CCI in regarding to inflammation and autophagy continues to evolve in both fundament and translation. However, few neuroprotective approaches have been successfully translated into the clinic due to its complex pathogenesis. Here, we reviewed the possible mechanisms of autophagy and inflammation, and we also discussed whether and how these factors affect CCI, which will help us understand the occurrence of CCI and find new ways to target autophagy to control brain tissue injury induced by excessive inflammation. The current study demonstrates that autophagy affects brain that drive or suppress inflammatory responses during CCI, which causes internal changes in CCI. This study may also potentially serve as a guide for future research and therapeutics of CCI, and it should be noted that monitoring autophagy may represent a key aspect of protecting the brain from CCI injury.

## Data Availability

Not applicable.
